# Self-Assembly of [3]Catenane and [4]Catenane Based on Neutral Organometallic Scaffolds

**DOI:** 10.3389/fchem.2021.805229

**Published:** 2021-12-13

**Authors:** Gui-Yuan Wu, Hong-Juan Zhu, Fang-Fang Pan, Xiao-Wei Sheng, Ming-Rui Zhang, Xianyi Zhang, Guangxin Yao, Hang Qu, Zhou Lu

**Affiliations:** ^1^ Anhui Province Key Laboratory of Optoelectronic Material Science and Technology, School of Physics and Electronic Information, Anhui Normal University, Wuhu, China; ^2^ State Key Laboratory of Physical Chemistry of Solid Surfaces, Collaborative Innovation Center of Chemistry for Energy Materials (iChEM) and College of Chemistry and Chemical Engineering, Xiamen University, Xiamen, China; ^3^ China Key Laboratory of Pesticide and Chemical Biology of Ministry of Education, College of Chemistry, Central China Normal University, Wuhan, China

**Keywords:** mechanically interlocked molecules, catenane, coordination-driven self-assembly, platinum–oxygen bond, metallacycle

## Abstract

Transition metal-mediated templating and self-assembly have shown great potential to construct mechanically interlocked molecules. Herein, we describe the formation of the bimetallic [3]catenane and [4]catenane based on neutral organometallic scaffolds *via* the orthogonality of platinum-to-oxygen coordination-driven self-assembly and copper(I) template–directed strategy of a [2]pseudorotaxane. The structures of these bimetallic [3]catenane and [4]catenane were characterized by multinuclear NMR {^1^H and ^31^P} spectroscopy, electrospray ionization time-of-flight mass spectrometry (ESI-TOF-MS), and PM6 semiempirical molecular orbital theoretical calculations. In addition, single-crystal X-ray analyses of the [3]catenane revealed two asymmetric [2]pseudorotaxane units inside the metallacycle. It was discovered that tubular structures were formed through the stacking of individual [3]catenane molecules driven by the strong π–π interactions.

## Introduction

[n]Catenanes are a class of mechanically interlocked molecules (MIMs) consisting of two or more macrocycles that are not covalently linked to each other (Nepogodiev et al., 1998; Evans et al., 2014) These fascinating molecules have attracted increasing attention not only because of their intriguing structures and topological importance but also as a result of their potential applications in molecular machines, biomaterials, and smart materials (Niu et al., 2009; Durola et al., 2014; Bruns et al., 2014; Fernando et al., 2016; Sawada, et al., 2016; Wu et al., 2017; Li et al., 2020; Gao et al., 2021; Lu et al., 2021). The interactions of molecular components which assist the formation of these interlocked molecules are advantageous to improve synthetic efficiency, thereby the synthesis of catenanes oftentimes utilizes the template-directed strategy which functions through molecular recognition and/or host–guest chemistry based on non-covalent interactions (Dietrich-Buchecker et al., 2003; Hao et al., 2020). These template-directed methods, including metal/organic ligand coordination, hydrophilic/hydrophobic interactions, anion templation, donor/acceptor interaction, and radical–radical interaction templating strategies, have been developed for the preparation of various topologically intriguing [n]catenanes (Kim, 2002; Sambrook et al., 2004; Shen et al., 2021).

Over the past 3 decades, coordination-driven self-assembly has become a well-established methodology for constructing a variety of supramolecular coordination complexes (SCCs) with well-defined shapes and sizes such as one-dimensional (1D) helices, two-dimensional (2D) polygons, and three-dimensional (3D) polyhedrons (Bhat et al., 2015; Wang et al., 2015; Chen et al., 2015; Chen and Yang 2018; Wu et al., 2018; Xiao et al., 2020; Wang et al., 2021; Wang et al., 2021). The strategy pattern is usually implemented *via* the combinatorial coordination between acceptor fragments with transition-metal and donor precursors with nitrogen/carboxylate. MIMs constructed by orthogonal or hierarchical self-assembly based on coordination-driven self-assembly and other binding motifs have attracted the increasing attention recently because of their high efficiency and strong pre-organization (Li et al., 2014; Lu et al., 2018). In 2020, we reported that the construction of the [3]catenane and molecular necklace based on the charged organometallic scaffold, by employing hierarchical self-assembly involving nitrogen–platinum coordination-driven self-assembly and Cu(I) template–directed strategy (Wu et al., 2020). It should be noted that there is another established way to construct metallacycles based on oxygen-to-platinum coordination-driven self-assembly that resulted in the formation of neutral supramolecular assemblies (Xu et al., 2016). However, the construction of novel catenanes with neutral metallacycles as main scaffolds through coordination-driven self-assembly have not been reported yet. Hence, the Cu(I)-bis(phenanthroline)s-based [2]pseudorotaxane donor fragments with dicarboxylate were a kind of excellent candidates for the oxygen-to-platinum coordination-driven self-assembly of [n]catenanes. Herein, we designed and synthesized a new 60^o^ [2]pseudorotaxane donor ligand from the dicarboxylate linear molecular axis and a 30-membered ring with the phenanthroline unit through the Cu(I) template–directed strategy. Subsequently, the [3]catenane with the neutral rhomboid scaffold and [4]catenane with the neutral triangular scaffold were successfully obtained via the formation of oxygen-to-platinum coordination bonds from the [2]pseudorotaxane donor ligand and the corresponding di-Pt(II) acceptors, respectively.

## Materials and Methods

All solvents were dried according to standard procedures, and all of them were degassed under N_2_ for 30 min before use. All air-sensitive reactions were carried out under an inert N_2_ atmosphere. The ^1^H and ^31^P NMR spectra were recorded on a Bruker 500 MHz spectrometer (^1^H: 500 MHz; ^31^P: 202 MHz) at 298 K. The ^1^H and ^31^P NMR chemical shifts were reported relative to the residual solvent signals. Coupling constants (*J*) were denoted in Hz and chemical shifts (*δ*) in ppm. Multiplicities were denoted as follows: s = singlet, d = doublet, m = multiplet, and br = broad. The CSI-TOF-MS spectra were acquired by using an AccuTOF CS mass spectrometer (JMS-T100CS, JEOL, Tokyo, Japan). Single crystal X-ray diffraction data were collected at room temperature on XtaLAB Synergy (Dualflex, HyPix). The X-ray single-crystal diffractometer was used to study Cu K_α_ (*λ* = 1.54184 Å) micro-focus X-ray sources (PhotonJet (Cu) X-ray Source). The raw data were collected and processed by CrysAlisPro software. The structures were solved by SHELXT with intrinsic phasing and refined on *F* ^2^ by full-matrix least-squares methods with SHELXL and OLEX2 used as GUI.

## Results and Discussion

### Synthesis and Characterization

**SCHEME 1:**
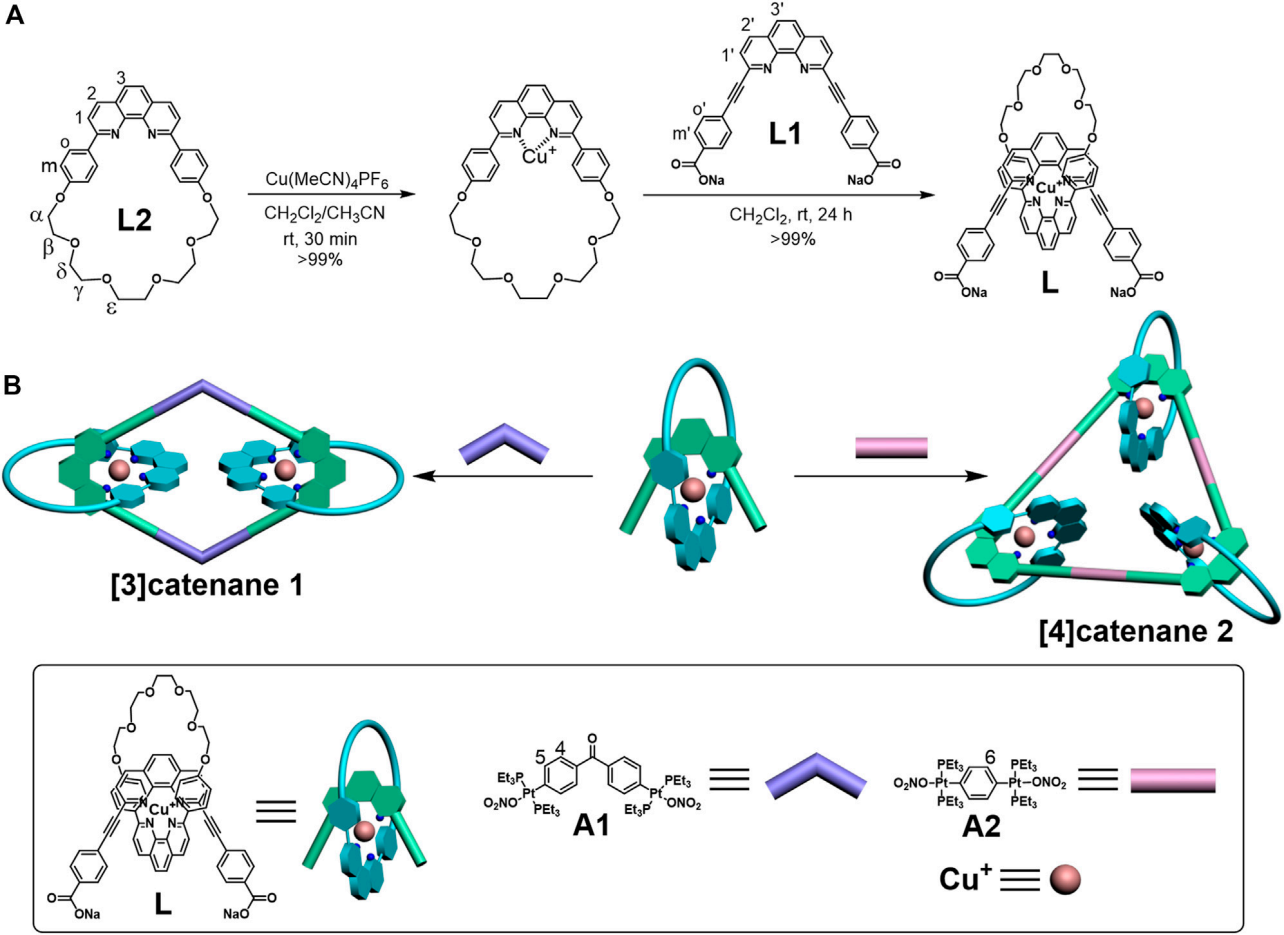
Graphical representation of the self-assembled donor **L (A)** and [3]catenane and [4]catenane **(B)**.

The hierarchical formation of the [3]catenane and [4]catenane was achieved via successive utilization of Cu(I) template–directed strategy and oxygen-to-platinum coordination-driven self-assembly (Scheme 1A). The 60° linear molecular axis **L1**, contained 1,10-phenanthroline (phen) and two carboxylate binding sites, can be easily synthesized according to the previous reports (Coskun et al., 2012). The [2]pseudorotaxane dicarboxylate donor **L** was quantitatively afforded between **L1** and macrocycle **L2** via the Cu(I) template–directed strategy. In a Schlenk flask, 1.0 equiv macrocycle **L2** (29.47 mg, 0.052 mmol) was dissolved under nitrogen in a 20-ml (V:V = 1:1) mixture of dichloromethane and acetonitrile. After addition of 1.0 equiv Cu(MeCN)_4_PF_6_ (19.38 mg, 0.052 mmol), the reaction was stirred at room temperature under nitrogen for 30 min. In a second Schlenk flask, 1.0 equiv **L1** (26.65 mg, 0.052 mmol) was dissolved in dichloromethane (20 ml) and cannula-filtered into the first solution. The solution was stirred under nitrogen at room temperature for an additional 24 h, followed by the removal of the solvent in vacuo to dryness to afford quantitatively L as a brown-red solid (yield = 66.29 mg, 99%). **L** did not obtain ^1^H NMR because its solubility is very poor in organic solvents.

**FIGURE 1:**
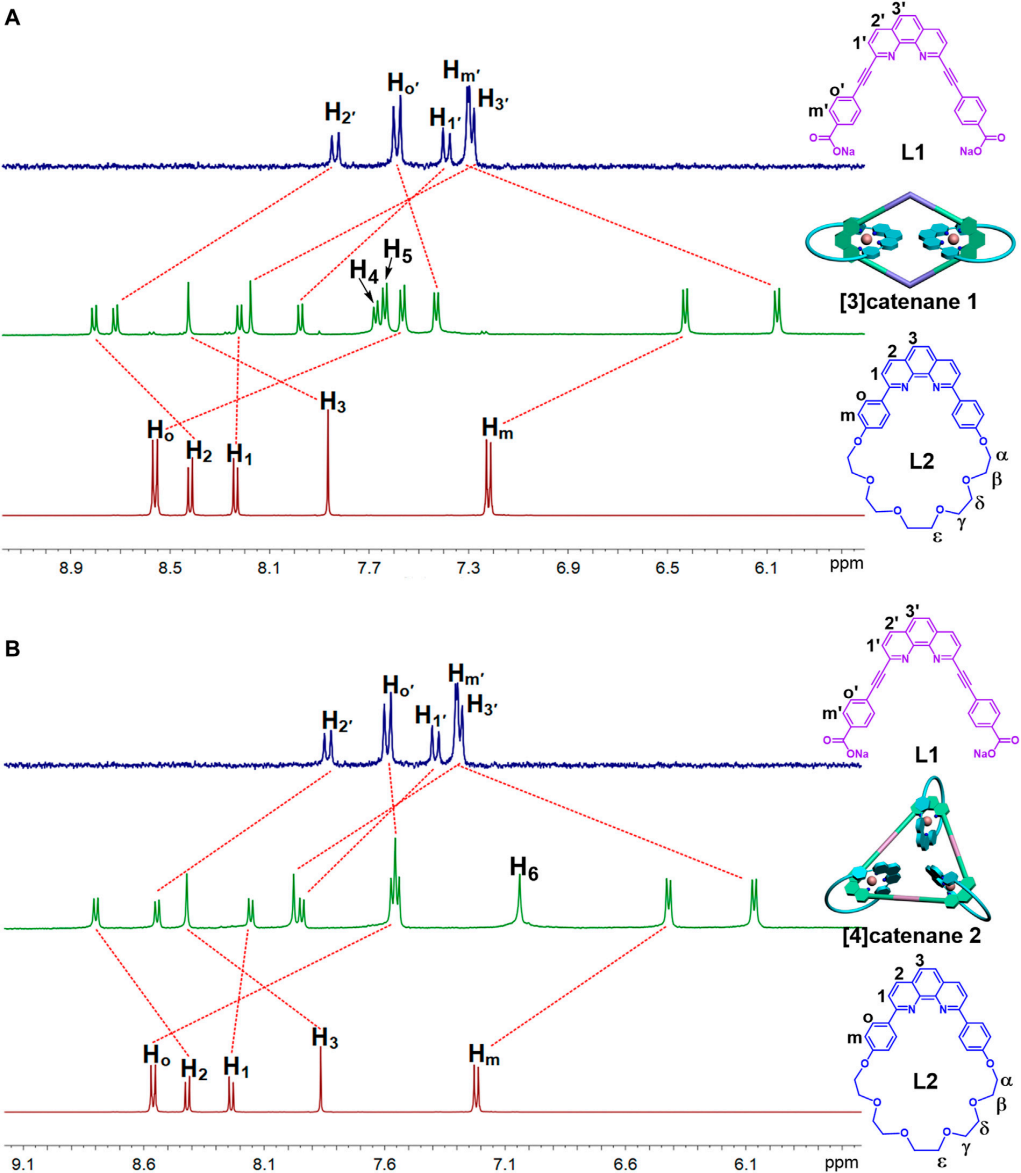
^1^H NMR spectra (500 MHz, 298 K) of the [3]catenane-**A1 (A)** and [4]catenane-**A2 (B)** in acetone-*d*_6_.

According to the coordination-driven self-assembly strategy, the [3]catenane **1** based on the rhomboidal scaffold was obtained by stirring donor **L** with an equimolar amount of a 120° di-Pt(II) acceptor **A1** in a 1:7.5 ratio in H_2_O/acetone at 50°C for 24 h (Figure 1) (Li et al., 2014; Wu et al., 2018; Hu et al.,. 2020). Similarly, the [4]catenane **2** with the triangular scaffold was formed with three [2]pseudorotaxanes **L** and three 180° di-Pt-(II) acceptors **A2** (Scheme 1B). The donor ligand **L** (8.85 mg, 6.87 μmol) and 120^o^ organoplatinum acceptor **A1** (8.02 mg, 6.87 μmol) were weighed accurately into a glass vial. A total amount of 3.75 ml acetone and 0.5 ml H_2_O were added into the vial, and the reaction solution was stirred at 50°C for 24 h. The PF_6_^−^ salt of **1** was synthesized by dissolving the NO_3_^−^ salts of **1** in acetone/H_2_O and adding a saturated aqueous solution of KPF_6_ to precipitate the product, which was collected by vacuum filtration (yield = 15.54 mg, 99%). ^1^H NMR (500 MHz, acetone-*d*_6_): *δ* 8.81 (H_2_, d, *J* = 8.2 Hz, 2H), 8.72 (H_2ʹ_, d, *J* = 8.2 Hz, 2H), 8.43 (H_3_, s, 2H), 8.22 (H_1_, d, *J* = 8.2 Hz, 2H), 8.18 (H_3ʹ_, s, 2H), 7.98 (H_1ʹ_, d, *J* = 8.2 Hz, 2H), 7.66 (H_4_, dd, *J* = 18.5, 7.9 Hz, 7H), 7.57 (H_5_, d, *J* = 8.4 Hz, 4H), 7.43 (H_o_, d, *J* = 7.9 Hz, 4H), 6.43 (H_m_, d, *J* = 8.0 Hz, 4H), 6.06 (H_mʹ_, d, *J* = 8.5 Hz, 4H), 3.87 (H_ε_, s, 4H), 3.78–3.72 (H_δ_, m, 4H), 3.64–3.57 (H_γ_, m, 4H), 3.48 (H_αβ_, dd, *J* = 22.4, 4.8 Hz, 8H), 1.66 (PCH_2_-, dd, *J* = 7.3, 3.6 Hz, 24H), 1.23 (-CH_3_, dt, *J* = 15.7, 7.7 Hz, 37H). ^31^P NMR (acetone-*d*_6_, 202 MHz): *δ* 16.51 ppm.

Following the preparation of **1** (Scheme 1), the self-assembly of the donor ligand **L** (9.86 mg, 7.66 μmol) with the 180^o^ organoplatinum acceptor **A2** (8.14 mg, 7.66 μmol) led to the formation of the pure [4]catenane **2** (16.53 mg, 99%). ^1^H NMR (500 MHz, acetone-*d*_6_): *δ* 8.80 (H_2_, d, *J* = 8.2 Hz, 2H), 8.54 (H_2ʹ_, d, *J* = 8.2 Hz, 2H), 8.42 (H_3_, s, 2H), 8.16 (H_1_, d, *J* = 8.2 Hz, 2H), 8.02–7.90 (H_1ʹ_, H_3ʹ_, m, 4H), 7.56 (H_o_, H_oʹ_, t, *J* = 8.5 Hz, 6H), 7.04 (H_6_, s, 4H), 6.42 (H_m_, d, *J* = 7.8 Hz, 4H), 6.07 (H_mʹ_, d, *J* = 8.3 Hz, 4H), 3.86 (H_ε_, s, 4H), 3.79–3.69 (H_δ_, m, 4H), 3.65–3.57 (H_γ_, m, 4H), 3.48 (H_αβ_, dd, *J* = 22.1, 4.7 Hz, 8H), 1.70 (PCH_2_-, dd, *J* = 22.7, 19.1 Hz, 24H), 1.24 (-CH_3_, ddd, *J* = 30.2, 15.2, 7.2 Hz, 36H). ^31^P NMR (acetone-*d*_6_, 202 MHz): *δ* 16.62 ppm. ESI-MS: m/z: 2036.40 [M-3PF_6_]^3+^.

**FIGURE 2:**
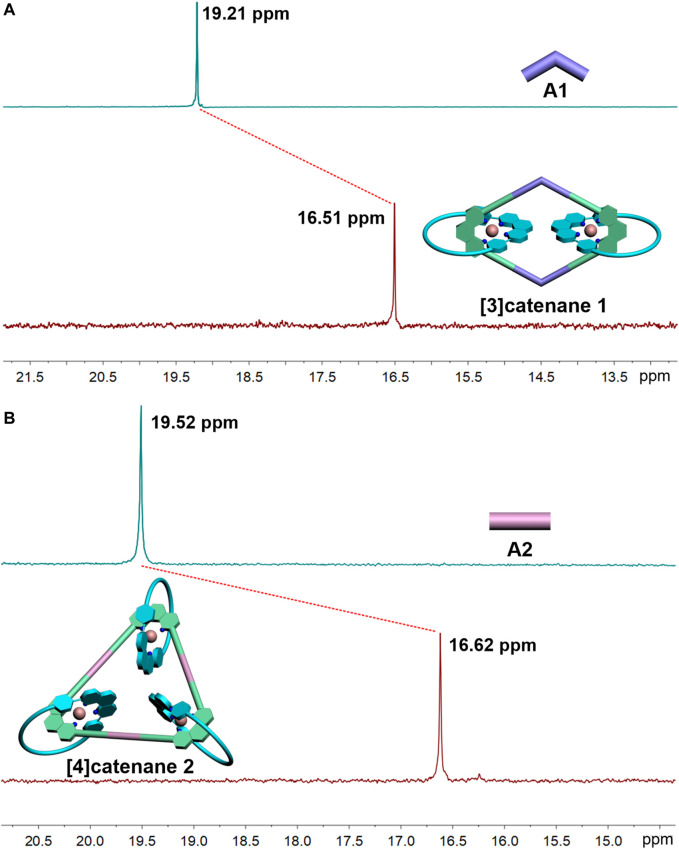
^31^P NMR spectra (500 MHz, 298 K) of the [3]catenane-**A1 (A)** and [4]catenane-**A2 (B)** in acetone-*d*_6_.

Multinuclear NMR (^1^H and ^31^P) analysis of the [3]catenane and [4]catenane revealed the formation of single, discrete assemblies. The most prominent features in ^1^H NMR spectra of the [3]catenane **1** and [4]catenane **2** were the obvious upfield shifts of the protons (**1**-H_o_: 8.55–7.56 ppm; **1**-H_m_: 7.22–6.43 ppm; **1**-Hoʹ: 7.59–7.43 ppm; **1**-H_mʹ_: 7.3–6.06 ppm; **2**-H_o_: 8.55–7.55 ppm; **2**-H_m_: 7.22–6.42 ppm; **2**-H_oʹ_: 7.59–7.55 ppm; **2**-H_mʹ_: 7.3–6.06 ppm) assigned to the phen moieties, which can be explained by the two orthogonally oriented phens around the Cu(I) ion being magnetically shielded by each other (Figure 1). The ^31^P NMR spectra of [3]catenane **1** and [4]catenane **2** displayed a sharp singlet (for **1**, ∆*δ*_**1**-**A1**_ = −2.7 ppm; for **2**, ∆*δ*_**2**-**A2**_ = −2.9 ppm) that shifted upfield from the signal of the starting platinum acceptor **A1** and **A2** (Figure 2) due to the electron back-donation from the platinum atoms. In addition, the structures of the [4]catenane **2** were further confirmed by ESI-TOF-MS, which allowed the assembly to remain intact to the maximum extent during the ionization process, while obtaining the high resolution required for isotopic distribution. For instance, the ESI-TOF-MS spectrum of **2** revealed signals that corresponded to charge states resulting from the loss of PF_6_^−^ counterions, [**2**-3PF_6_]^3+^, in which **2** represents the intact assembly (Supplementary Figure S2).

### Single Crystal X-Ray Diffraction Characterization and Theoretical Calculations

**FIGURE 3:**
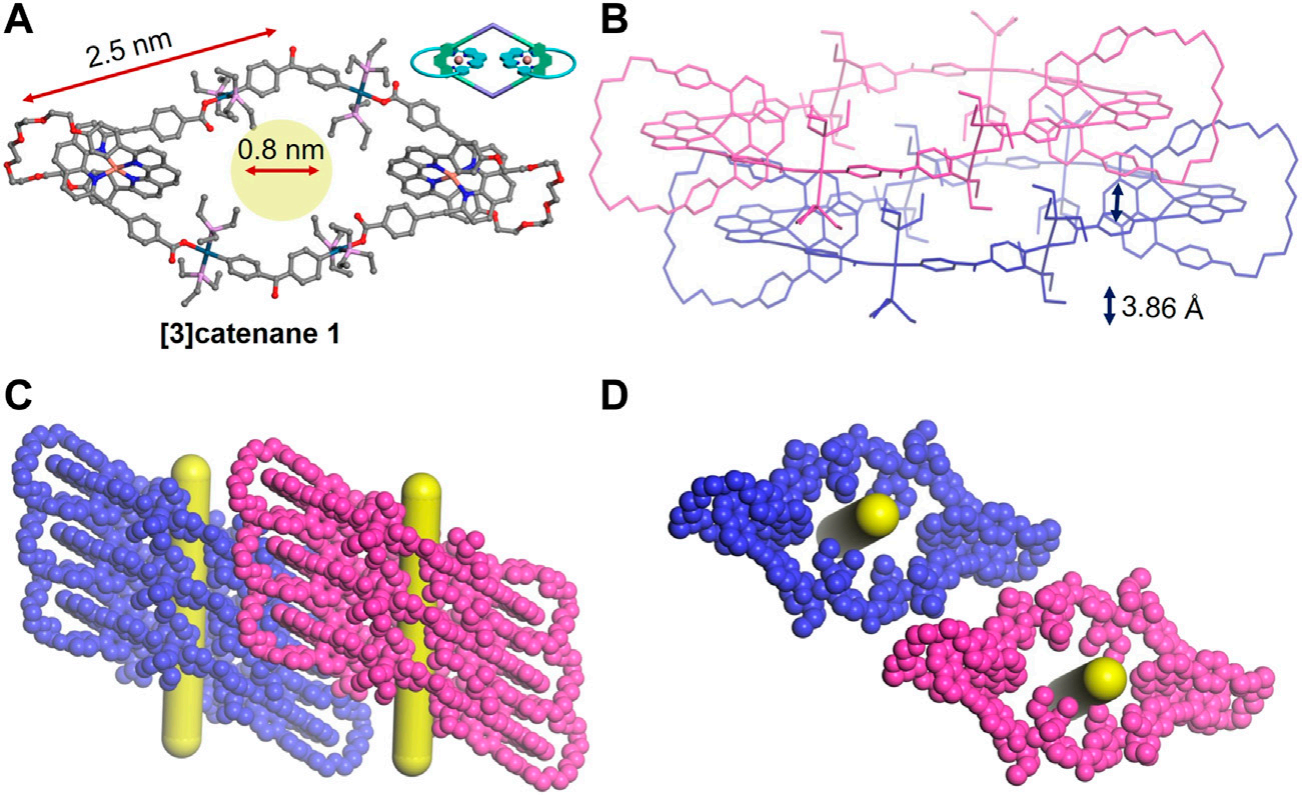
Single-crystal structures **(A)**, two closely packed structures **(B)**, side view **(C),** and top view **(D)** of 3D packing structures of the [3]catenane **1**. Hydrogen atoms and PF_6_^−^ anions are omitted for clearance.

The mechanically interlocked structures of the [3]catenane **1** were clearly demonstrated by X-ray crystallographic analysis (Figure 3). The single crystals of **1** were grown by slow evaporation of its dichloromethane solution. Mesomeric **1** crystallizes in the P-1 space group with two asymmetric [Cu(phen)_2_]^+^ units, threading two polyether phenanthroline macrocycles onto the main Pt(II)-O-coordinated rhombic metallacycles (Figure 3A). The exterior length of **1** is approximately 2.5 nm, resulting in the large cavity with a diameter of approximately 0.8 nm. In addition, the phenyl ring of adjacent molecules exhibited the strong π–π interactions with a centroid-to-centroid distance of 3.86 Å (Figure 3B), thus leading to a liner packing of rhombic motifs and generating an interconnected 1D pore channel (Figures 3C,D). Thus, such kinds of catenanes based on organometallic skeletons are expected to be useful in host–guest chemistry.

**FIGURE 4:**
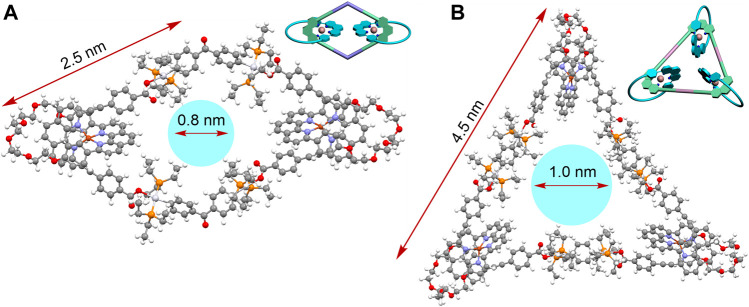
Geometric structures of the [3]catenane **1 (A)** and [4]catenane **2 (B)**, which were optimized at the PM6-DH + level by the semi-empirical quantum chemistry package.

To better understand the spatial structures of these catenanes, the PM6 semi-empirical quantum chemistry method (PM6-DH+) was employed to obtain the optimized geometry of the [3]catenane **1** and [4]catenane **2** (Stewart, 2007). The molecular simulation disclosed the cavity diameter of [3]catenane **1** and [4]catenane **2** to be around 0.8 and 1.0 nm and the exterior length to be approximately 2.5 and 4.5 nm, respectively (Figure 4). This finding indicated that the simulation results of the [3]catenane **1** were consistent with the single crystal data. In addition, the [4]catenane based on the Pt(II)-O bonds organometallic scaffold have a relatively larger internal cavity size and exterior length than [4]catenane based on the Pt(II)-N bonds organometallic scaffold (for the [4]catenane, cavity diameter: ca. 0.8 nm; exterior length: ca. 3.7 nm) (Wu et al., 2020). These observations were attributed to the different lengths of building blocks because the dicarboxylate donor **L1** was featured with longer length than that of the dipyridine donor in the [4]catenane based on the Pt(II)-N-bonded organometallic scaffold.

## Conclusion

In conclusion, we have shown the highly efficient construction of the [3]catenane and [4]catenane via hierarchical assembly strategy wherein oxygen-to-platinum coordination-driven self-assembly and copper(I) template–directed strategy of a 1,10-phenanthroline-based [2]pseudorotaxane comprising 30-membered rings and 60° dicarboxylate donors. Multinuclear NMR {^1^H and ^31^P} spectroscopy, electrospray ionization time-of-flight mass spectrometry (ESI-TOF-MS), and the PM6 semiempirical molecular orbital calculations unambiguously supported for molecular compositions. In the case of the [3]catenane, the structures of the assemblies have been established by X-ray crystallography. The crystallographic studies revealed two asymmetric [Cu(phen)_2_]^+^ units inside the [3]catenane and tubular structures through the stacking of the individual [3]catenane driven by the strong π–π interactions. Such hierarchical assembly strategy, which successively used highly efficient oxygen-to-platinum coordination-driven self-assembly and the template-directed strategy, may provide insights into the construction of other topologically complex supermolecules with well-defined structures.

## Data Availability

The original contributions presented in the study are publicly available. This data can be found here: https://zenodo.org/record/5748670#.YaiB8MiEzWE.
