# Supplementary material for: Self-Assembly of [3]Catenane and [4]Catenane Based on Neutral Organometallic Scaffolds
Source: Front Chem. 2021 Dec 13;9:805229. doi: 10.3389/fchem.2021.805229 (PMC8710481; doi:10.3389/fchem.2021.805229)
Supplement: Supplementary file 1 [file DataSheet1.docx]

Self-Assembly of [3]Catenane and [4]Catenane Based on Neutral Organometallic Scaffolds

Gui-Yuan Wu*^1^, Hong-Juan Zhu^1^, Fang-Fang Pan^3^, Xiao-Wei Sheng^1^, Ming-Rui Zhang^1^, Xianyi Zhang^1^, Guangxin Yao^1^, Hang Qu*^2^, Zhou Lu*^1^

^1^Anhui Province Key Laboratory of Optoelectronic Material Science and Technology, School of Physics and Electronic Information, Anhui Normal University, Wuhu, China

^2^State Key Laboratory of Physical Chemistry of Solid Surfaces, Collaborative Innovation Center of Chemistry for Energy Materials (iChEM) and College of Chemistry and Chemical Engineering, Xiamen University, Xiamen 361005, China

^3^China Key Laboratory of Pesticide and Chemical Biology of Ministry of Education, College of Chemistry, Central China Normal University, Wuhan 430079, China

Correspondence:

Gui-Yuan Wu, Zhou Lu, Hang Qu

wgy@ahnu.edu.cn (G-Y Wu); zhoulu@ahnu.edu.cn (Z Lu); quhangxmu@qq.com (H Qu)

**Table of Contents**

1. Materials and methods………………………………………………………………….….….........S2

2. Synthetic experimental details and characterizations of new compounds ……………………....….S2

3. Single-crystal X-ray diffraction………..……………………………………………….….….........S4

4. ^1^H and ^31^P NMR and mass spectra of all new compounds………………………………………….S5

5. References...........………………………………………………….……………………..……….S7

**1. Synthetic experimental details and characterizations of new compounds**

**Scheme S1.** The synthetic procedure for the [3]catenane **1**.

**Synthesis of [3]catenane 1.** Self-assembly of [3]catenane **1** from donor **L** and diplatinum acceptor **A1**. The donor ligand **L** (8.85 mg, 6.87 μmol) and 120^o^ organoplatinum acceptor **A1** (8.02 mg, 6.87 μmol) were weighed accurately into a glass vial. To the vial was added 3.0 mL acetone and 0.4 mL H_2_O, and the reaction solution was stirred at room temperature for 24 hours. The PF_6_^-^ salt of **1** was synthesized by dissolving the NO_3_^-^ salt of **1** in acetone/H_2_O and adding a saturated aqueous solution of KPF_6_ to precipitate the product, which was collected by vacuum filtration. Yield: 15.54 mg, 99%.

**Scheme S2.** The synthetic procedure for the [4]catenane **2**.

**Synthesis of [4]catenane 2.** Self-assembly of [4]catenane 2 from donor **L** and diplatinum acceptor **A2**. The donor ligand **L** (9.86 mg, 7.66 μmol) and 180^o^ organoplatinum acceptor **A2** (8.14 mg, 7.66 μmol) were weighed accurately into a glass vial. To the vial was added 2.0 mL acetone and 0.4 mL H_2_O, and the reaction solution was stirred at room temperature for 24 hours. The PF_6_^-^ salt of **2** was synthesized by dissolving the NO_3_^-^ salt of **2** in acetone/H_2_O and adding a saturated aqueous solution of KPF_6_ to precipitate the product, which was collected by vacuum filtration. Yield: 16.53 mg, 99%.

**2. Single-crystal X-ray diffraction.**

Single crystal X-ray diffraction data were collected at room temperature on XtaLAB Synergy (Dualflex, HyPix). X-Ray single crystal diffractometer using Cu K_α_ (λ = 1.54184 Å) micro-focus X-ray sources (PhotonJet (Cu) X-ray Source). The raw data were collected and reduced by CrysAlisPro software. The structures were solved by the SHELXT^1^ with Intrinsic Phasing and refined on *F*^2^ by full-matrix least-squares methods with the SHELXL^1^ and OLEX2^2^ was used as GUI.

Refinement details: All non-hydrogen atoms were refined anisotropically. Hydrogen atoms were placed at calculated positions using the riding model and refined isotropically. The instructions AFIX 23 and AFIX 43 were used for the hydrogen atoms on the secondary -CH_2_- and the aromatic C-H, respectively, with the parameter of U_iso_ = 1.2 U_eq_. The instruction AFIX 33 was used for the hydrogen atoms on the terminal -CH_3_ groups with the parameter of Uiso=1.5 Ueq. Nevertheless, the flexible alkyl chains are expected to be highly disordered. Therefore, necessary Shelx restraints (i.e., DELU, SIMU and DFIX) were applied to the alkyl chains to result in a reasonable model. Specifically, the anisotropic displacement parameters of disordered atoms in alkyl chains were restrained to be equal within an effective standard deviation of 0.001 using the DELU command.

Although absorption correction has been done, there are two A alerts about calculated positive residual density on Pt in checkcif report. However, there is no chemically sensible species corresponding to this residual density peak. It can be consequence of thermal restraints in the refinement.

A satisfactory disorder model for the solvent molecules was not found, therefore the OLEX_2_ Solvent Mask routine (similar to PLATON/SQUEEZE) was used to mask out the disordered density.

**3. ^1^H and ^31^P NMR and mass spectra of all new compounds**

**Figure S1.** Theoretical (top) and experimental (bottom) ESI-TOF-MS spectra of [4]catenane **2**.


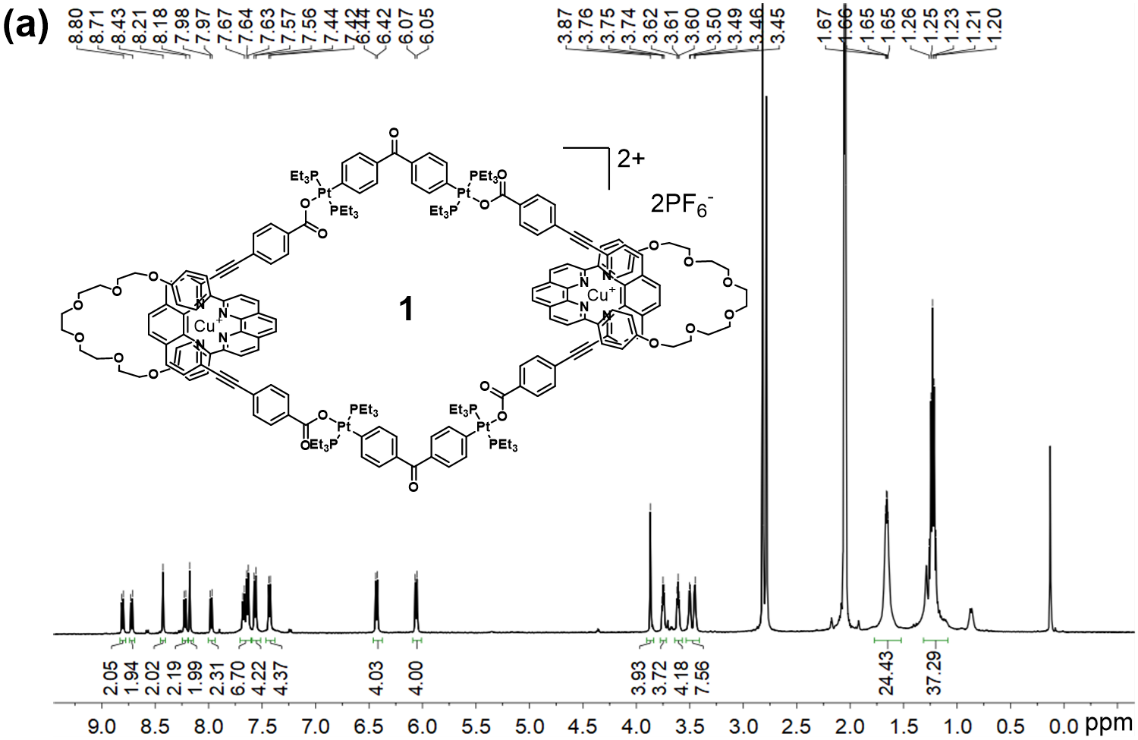


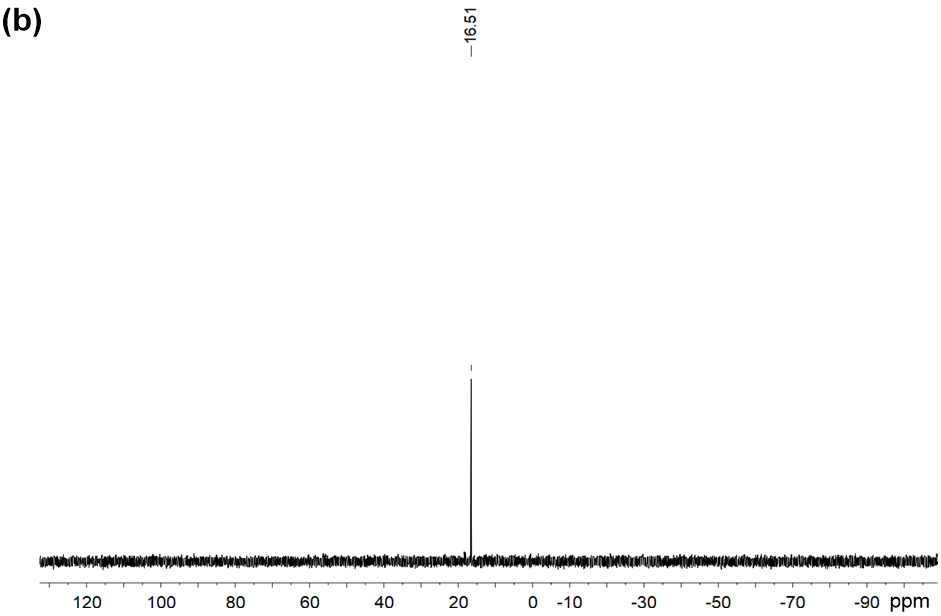


**Figure S2.** (a) ^1^H NMR spectrum (500 MHz, acetone-*d*_6_, 298K), (b) ^31^P NMR spectrum (202 MHz, acetone-*d*_6_, 298K) of [3]catenane **1**.


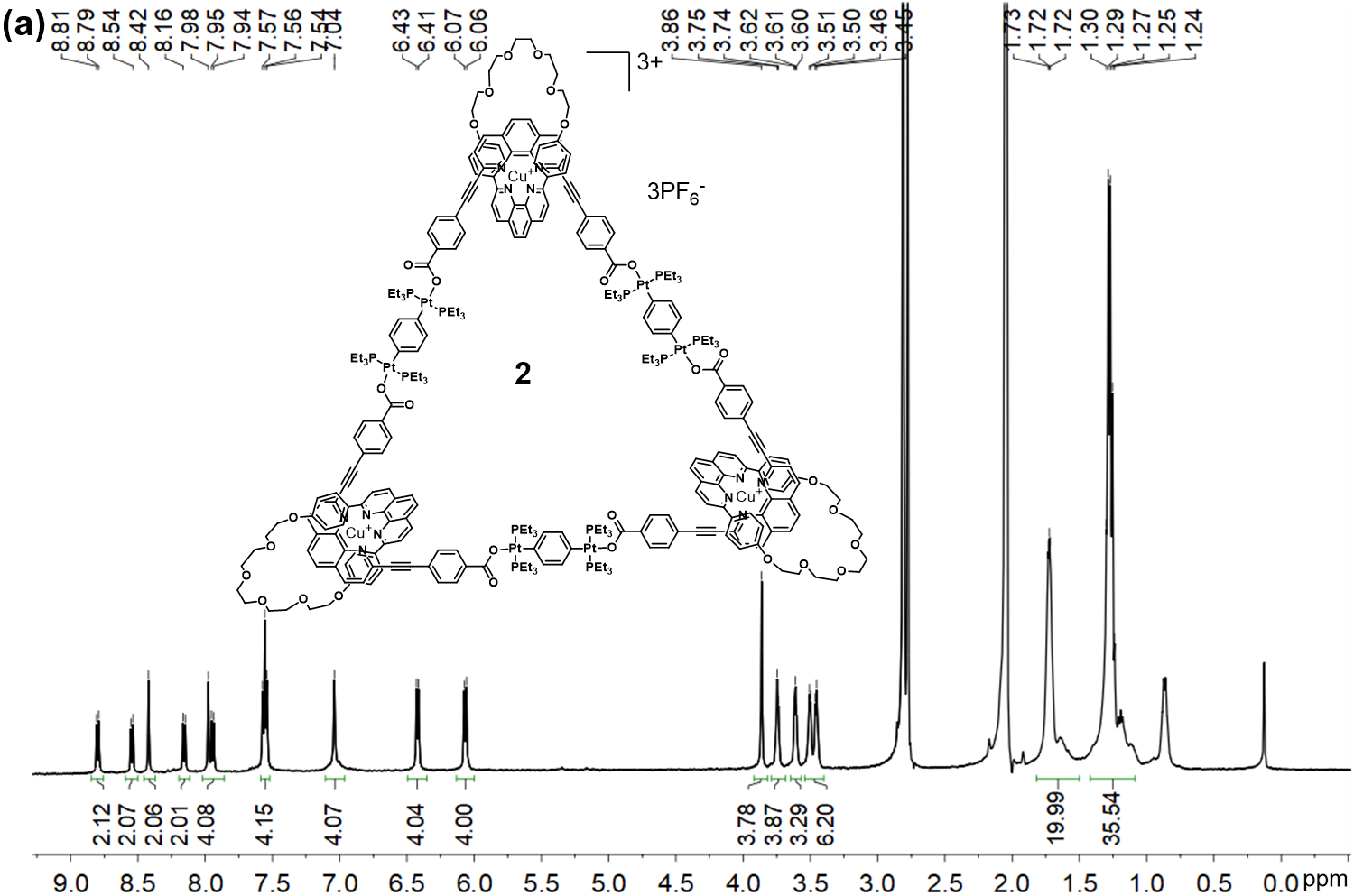


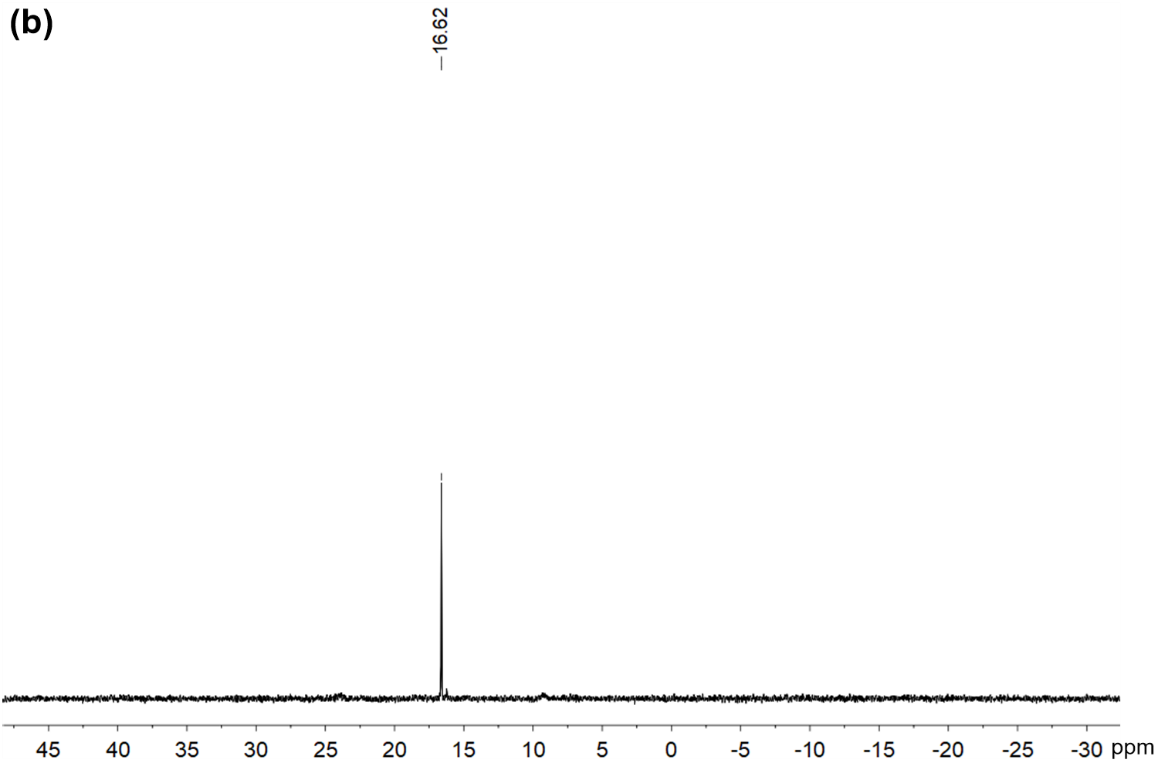


**Figure S3.** (a) ^1^H NMR spectrum (500 MHz, acetone-*d*_6_, 298K), (b) ^31^P NMR spectrum (202 MHz, acetone-*d*_6_, 298K) of [4]catenane **2**.

**4. Reference**

1. Sheldrick, G. M. *Acta Crystallogr. Sect. C*, **2015**, *71*, 3.
2. Dolomanov, O. V.; Bourhis, L. J.; Gildea, R. J. K.; Puschmann, H. J. *Appl. Crystallogr*. **2009**, *42*, 339.
